# Ellagic Acid Increases Stress Resistance via Insulin/IGF-1 Signaling Pathway in *Caenorhabditis elegans*

**DOI:** 10.3390/molecules27196168

**Published:** 2022-09-20

**Authors:** Shuju Bai, Yaoru Yu, Lu An, Wenbo Wang, Xueqi Fu, Jing Chen, Junfeng Ma

**Affiliations:** National Engineering Laboratory for AIDS Vaccine, School of Life Sciences, Jilin University, Changchun 130012, China

**Keywords:** ellagic acid, insulin/IGF-1 signaling pathway, ultraviolet radiation, *Caenorhabditis elegans*, ROS

## Abstract

Ellagic acid is a natural polyphenol found in various fruits and vegetables. Numerous studies have shown that ellagic acid has beneficial effects on human health. In this study, we investigated the stress resistant action of ellagic acid in *Caenorhabditis elegans* (*C. elegans*). Notably, 50 μM ellagic acid prolonged the lifespan of *C. elegans* by 36.25%, 36.22%, 155.1%, and 79.07% under ultraviolet radiation stress, heat stress, oxidative stress, and *Pseudomonas aeruginosa* infection stress, respectively. Furthermore, the mechanism by which ellagic acid reduces the damage caused by ultraviolet radiation in *C. elegans* was explored. Ellagic acid could significantly induce the nucleus translocation of DAF-16 and, thereby, activate a series of target genes to resist ultraviolet radiation stress. Moreover, ellagic acid also significantly increased the expression of SOD-3 by 3.61 times and the activity of superoxide dismutase by 3.70 times to clean out harmful reactive oxygen species in *C. elegans* exposed to ultraviolet radiation stress. In both *daf-16* mutant and *daf-2*; *daf-16* double-mutant worms exposed to ultraviolet radiation, ellagic acid could no longer prolong their lifespan. These results indicate that ellagic acid plays an important role in resisting ultraviolet radiation stress in *C. elegans,* probably in an insulin/IGF-1 signaling pathway-dependent way.

## 1. Introduction

Organisms inevitably exist in a multiple-stress environment. The progression of many diseases is related to the inadequate defense of the organism against external stress. There are many stresses in human daily life, such as ultraviolet (UV) radiation stress [1], heat stress [2], and oxidative stress [3]. These stimuli can disrupt the balance between the body’s oxidative and antioxidant systems, resulting in the production and accumulation of large amounts of reactive oxygen species (ROS) and the excess ROS can ultimately lead to oxidative reactions that affect human health [4]. For instance, long-term exposure to UV radiation has a detrimental effect on the skin, causing erythema, burns, and, eventually, skin cancer [5,6,7]. Furthermore, oxidative stress is the molecular basis of many diseases, including diabetes, arteriosclerosis, neurodegenerative diseases, stroke, and cataracts [8]. Therefore, exploring an effective way to resist the damage of stress is vital for the daily life of humans.

Numerous studies have shown that various natural compounds derived from plants can effectively prevent the damage caused by stress [9,10]. As a secondary polyphenolic metabolite, ellagic acid is a dimeric derivative of gallic acid found in various vegetables and fruits [11]. To date, ellagic acid has attracted more and more attention for its potential health benefits; e.g., antioxidant, anti-inflammatory, antiviral, and anticancer effects [12,13]. Several studies have also demonstrated that ellagic acid is effective against a variety of pathogens, including bacteria, fungi, and parasites [14,15,16]. In mice, liposomal ellagic acid attenuated cyclophosphamide-induced toxicity and eliminated systemic novel cryptococcal infections in leukocytopenic mice [17]. Moreover, ellagic acid can also reduce the damage caused by UV radiation by mediating the inflammatory cascade or reducing oxidative stress in skin cells [18,19,20]. However, the specific pathways through which ellagic acid resists stress damage in organisms have not been determined.

*Caenorhabditis elegans* (*C. elegans*) is a simple invertebrate roundworm with a complete physiological structure. With an increase in age, the movement capacity and physiological indexes of *Caenorhabditis elegans* show similar declines to those of mammals and humans [21]. In addition, the properties to the human genome, such as short life, quick fertility, and high homology, make *C. elegans* an excellent model organism for studying aging-related diseases. Moreover, *C. elegans* is also suitable for characterizing the damage caused by many stresses, as stress damage can be well-reflected by judging the survival of nematodes. Here, using *C. elegans*, the effects of ellagic acid on resisting several harmful stresses were explored. In *C. elegans,* DAF-16 is homologous to human FOXO and is downstream of the insulin/IGF-1 signaling pathway [22]. Usually, DAF-16 is supposed to function in the nucleus as a transcription factor that activates the expression of genes essential for longevity and stress resistance [23]. Hence, the mechanism by which ellagic acid increased the resistance of stress in *C. elegans* was further focused on, especially its relation to DAF-16.

## 2. Results

### 2.1. Ellagic Acid Increases the Stress Resistance of Nematodes

**Figure 1:**
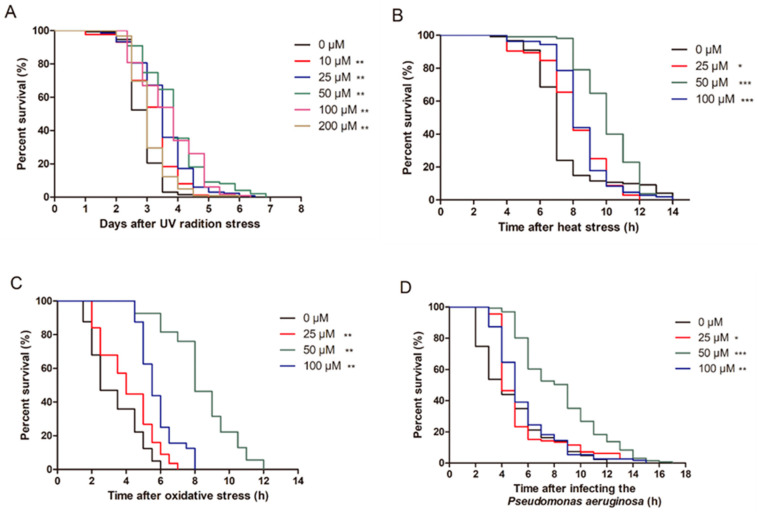
The effects of ellagic acid on the wild-type nematodes N_2_ under various stresses. (**A**) L4-stage worms were cultured on NGM plates containing 0, 10, 25, 50, 100, and 200 μM ellagic acid for two days and then exposed to a UV radiation dose of 1000 J/cm^2^, *n* > 100. (**B**) Survival curves of the nematodes exposed to 0, 25, 50, and 100 μM ellagic acid under thermal stress of 35 °C, *n* > 100. (**C**) L4-stage worms were cultured on NGM plates containing different concentrations of ellagic acid (0, 25, 50, and 100 μM) for two days. After being exposed to H_2_O_2_-induced oxidative stress, the survival curves of the nematodes were observed every 0.5 h, *n* > 100. (**D**) Survival curves of the nematodes exposed to *Pseudomonas aeruginosa* infection with treatments of 0, 25, 50, and 100 μM ellagic acid, *n* > 100. *p* < 0.5 (*), *p* < 0.01 (**), and *p* < 0.001 (***).

To determine the effect of ellagic acid on stress resistance, the worms were cultured on NGM agar plates containing 0, 10, 25, 50, 100, and 200 μM ellagic acid, and their lifespan was measured after being exposed to UV radiation. Under UV radiation stress, ellagic acid significantly increased the average lifespan of the nematodes. Compared to the control nematodes, 10, 25, 50, 100, and 200 μM ellagic acid significantly prolonged the lifespan of the nematodes by 11.39%, 23.33%, 36.25%, 29.83%, and 7.76%, respectively (Figure 1A). Due to the effect of ellagic acid extending the lifespan of worms exposed to UV radiation being more obvious at 25, 50, and 100 μM, these three concentrations of ellagic acid were selected to perform subsequent experiments.

Furthermore, a range of stress assays (thermal stress, oxidative stress, and *Pseudomonas aeruginosa* infection stress) in *C. elegans* were also performed to determine the effects of ellagic acid on the resistance to other stresses. When examining thermal stress (35 °C), the mean lifespan of the worms treated with 25, 50, and 100 μM ellagic acid was significantly extended by 9.32%, 36.22%, and 14.19% versus the control, respectively (Figure 1B). Compared to the control *C. elegans*, 25, 50, and 100 μM ellagic acid also significantly prolonged the lifespan of N_2_ worms under oxidative stress of H_2_O_2_ by 24.4%, 155.1%, and 76%, respectively. Therefore, ellagic acid at 50 μM had the highest resistance to oxidative stress in the nematodes (Figure 1C). Similarly, ellagic acid also significantly extended the lifespan of the worms infected with *Pseudomonas aeruginosa,* while 50 μM ellagic acid was more effective in resisting *Pseudomonas aeruginosa* infection, which prolonged the lifespan of the N_2_ worms by 79.07% (Figure 1D).

### 2.2. Ellagic Acid Upregulates the Expression of Genes Involved with Stress Responses

**Figure 2:**
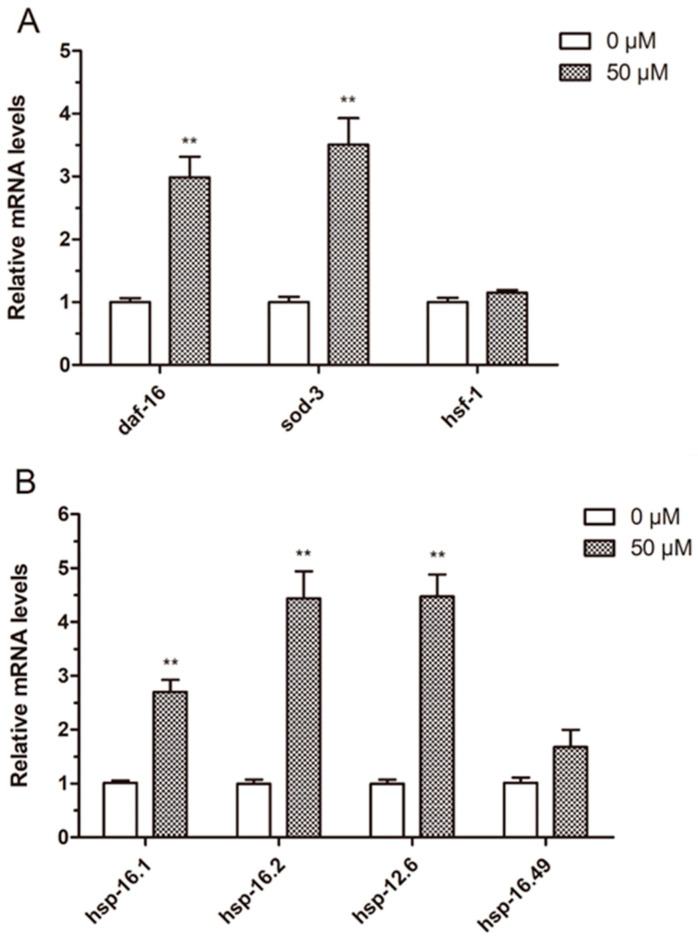
The effects of ellagic acid on the expression of genes related to the stress response in worms exposed to UV radiation. (**A**) The expression of the *daf-16*, *sod-3*, and *hsf-1* genes was measured using qPCR, *n* > 500. (**B**) The expression of small heat shock protein genes (*hsp-16.1*, *hsp-16.2*, *hsp-16.49*, and *hsp-12.6*) was determined with qPCR, *n* > 500. The data are shown as the mean ± SD. *p* < 0.01 (**).

As UV radiation was more stable and convenient, with 50 μM ellagic acid being more effective in resisting various stresses, UV radiation and ellagic acid at 50 μM were selected to determine the mechanism by which ellagic acid increased the stress resistance in nematodes. First, the expression of several genes related to the stress response, including *daf-16*, *sod-3*, *hsf-1*, *hsp-16.1*, *hsp-16.2*, *hsp-16.49*, and *hsp-12.6*, were measured by qPCR. Compared to the control group, the expression levels of *daf-16* and *sod-3* in those nematodes treated with ellagic acid were significantly increased by 2.97 and 3.46 times, respectively. However, the expression level of *hsf-1* showed no obvious difference between the control and ellagic acid-treated worms (Figure 2A). Moreover, as shown in Figure 2B, the expression levels of *hsp-16.1*, *hsp-16.2*, and *hsp-12.6* were also upregulated in the nematodes exposed to 50 μM ellagic acid. Nevertheless, the expression of *hsp-16.49* in the worms was not affected by the treatment with ellagic acid.

### 2.3. Ellagic Acid Induces DAF-16 Nucleus Localization and Decreases the Oxidative Stress Level

**Figure 3:**
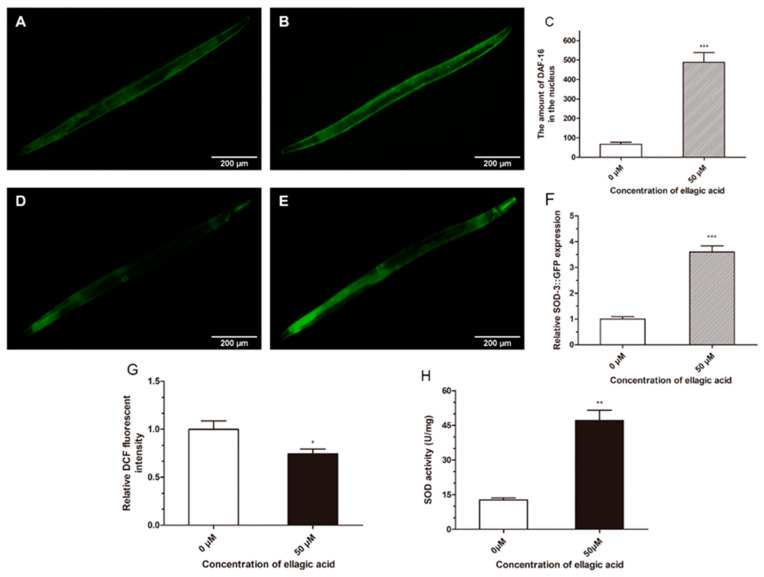
The effects of ellagic acid on the DAF-16 distribution and oxidative indices in the worms exposed to UV radiation. DAF-16 localization in the nucleus of the TJ356 control nematodes (**A**) and the ellagic acid treatment group (**B**) was measured using a fluorescence microscope under 10× objective, *n* > 15. (**C**) The amount of DAF-16 in the nucleus was calculated using ImageJ software. Each column was represented with the mean ± SD. The SOD-3 expression in the CF1553 worms of the control group (**D**) and the ellagic acid treatment group (**E**) was observed with a fluorescent microscope under a 10× objective, *n* > 15. (**F**) ImageJ was used to determine the fluorescence intensity of SOD-3 expression in the nematodes. Each column shows the mean ± SD. (**G**) The measurement of the ROS level was achieved by DCFH-DA fluorescence staining, *n* > 120. (**H**) The SOD activity was determined using a SOD assay kit, *n* > 500. Each column is represented by the mean ± SD. *p* < 0.5 (*), *p* < 0.01 (**), and *p* < 0.001 (***).

Under UV radiation, the effect of ellagic acid on DAF-16 distribution was detected using TJ356 transgenic worms, which can express the DAF-16::GFP protein. As shown in Figure 3A–C, ellagic acid significantly induced DAF-16 nucleus translocation of the nematodes compared to the control group. DAF-16 nucleus translocation can inevitably activate the expression of downstream genes and proteins [24]. SOD-3, one of the important downstream proteins of DAF-16, plays an important role in preventing oxidative damage [25]. Hence, the transgenic worm strain CF1553 was selected to determine the effect of ellagic acid on the expression of SOD-3 under UV radiation stress, as it can express the SOD-3::GFP protein. The results indicated that ellagic acid significantly increases the expression of SOD-3 by 3.61 times versus the control worms (Figure 3D–F).

Furthermore, several physiological indices (e.g., ROS contents and SOD activity) related to oxidative stress were also measured. The DCFH-DA reagent was used to detect the ROS level in nematodes exposed to UV radiation stress. The ROS level, indicated by the DCF fluorescence intensity, was significantly decreased by 24.3% in the worms treated with 50 μM ellagic acid compared to the control group (Figure 3G). Subsequently, a SOD activity assay kit was used to determine the SOD activity in the worms exposed to UV radiation stress. As shown in Figure 3H, the SOD activity was also significantly increased by 3.70 times in the nematodes treated with ellagic acid versus the control group.

### 2.4. Ellagic Acid Increases the UV Radiation Stress Resistance in the Nematodes through the Insulin/IGF-1 Pathway

**Figure 4:**
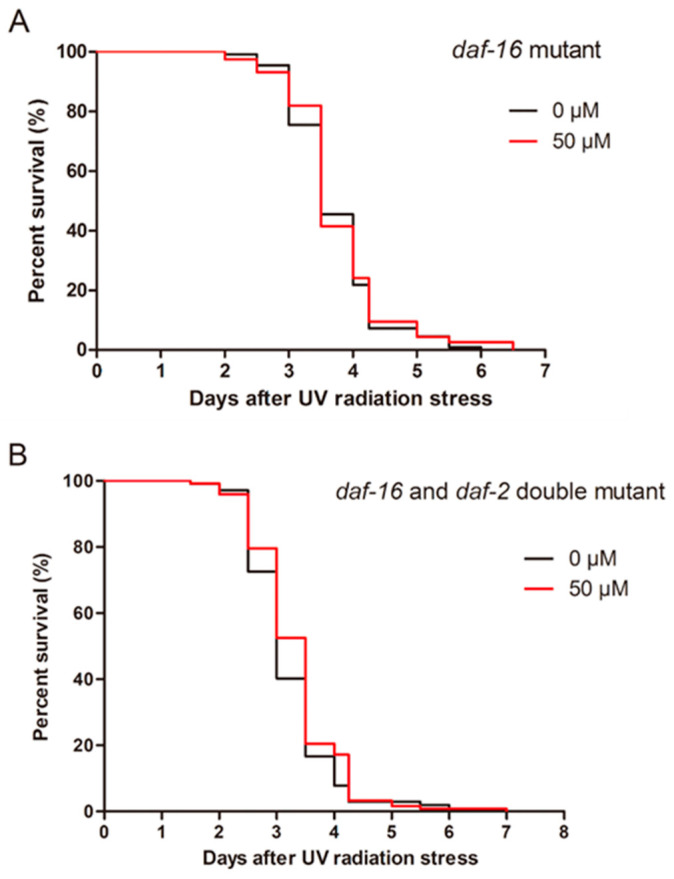
The effects of ellagic acid on the *daf-16* mutant worms (CF1308) and *daf-2* and *daf-16* double-mutant strains (CF1588) exposed to UV radiation stress. (**A**) The *daf-16* mutant worms CF1308 in the L4-stage were transferred onto NGM agar plates with 50 μM ellagic acid or without ellagic acid for two days. Then, the worms were exposed to a dose of 1000 J/cm^2^ of UV radiation, *n* > 100. (**B**) The survival of the *daf-2* and *daf-16* double-mutant nematodes exposed to UV radiation with 50 μM ellagic acid or without ellagic acid treatment, *n* > 100.

Related research has long held that the insulin/IGF-1 signaling pathway is an important signaling pathway regulating the lifespan and stress resistance of nematodes [26,27]. *daf-2*, which encodes a hormone receptor similar to the insulin and IGF-1 receptors, and *daf-16,* encoding a FOXO-transcription factor, are respectively located upstream and downstream in the insulin/IGF-1 signaling pathway [28,29]. Therefore, the effects of ellagic acid were assessed using *daf-16* mutant worms (CF1308) and *daf-2* and *daf-16* double-mutant worm strains (CF1588) under UV radiation stress. The results showed that ellagic acid did not prolong the average lifespan of the *daf-16* mutant worms CF1308 after exposure to UV radiation (Figure 4A). Similarly, in the *daf-2* and *daf-16* double-mutant nematodes CF1588, 50 μM ellagic acid could not extend the average lifespan of the nematodes under radiation (Figure 4B). Taken together, ellagic acid resists the UV radiation stress in nematodes via the insulin/IGF-1 signaling pathway-dependent manner.

### 2.5. Effect of Ellagic Acid on the Health Indexes of C. elegans

**Figure 5:**
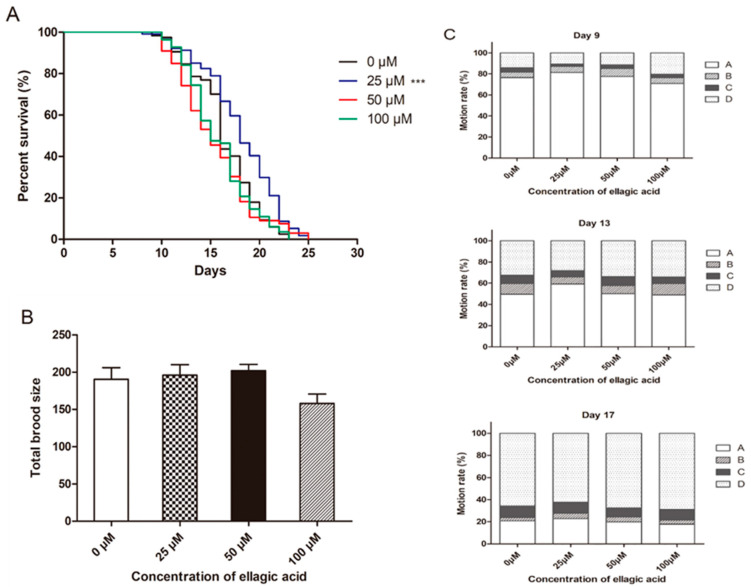
The effects of ellagic acid on the health indexes of the wild-type nematodes N_2_. L4-stage worms were transferred onto NGM agar plates containing different concentrations of ellagic acid (0, 25, 50, and 100 μM). (**A**) The survival analysis of worms at different concentrations of ellagic acid, *n* > 100. (**B**) Total brood size showed the total number of eggs laid by each worm, *n* > 10. Each column represents the mean ± SD. (**C**) The motility of the nematodes was observed respectively on days 9, 13, and 17. The graph represents the ratio of different movement states in the nematodes treated with or without ellagic acid, *n* > 100. *p* < 0.001 (***).

To determine the effect of ellagic acid on the lifespan, fertility, and movement capacity of nematodes, the survival time, total brood size, and motility of nematodes fed different concentrations of ellagic acid were also assessed. As shown in Figure 5A, 25 μM ellagic acid significantly extended the mean lifespan of the nematodes by 9.7% compared to the control group. However, the average lifespan of the worms was not significantly different between nematodes that underwent treatments of 50 and 100 μM ellagic acid and the control group. On the contrary, based on the counting assay of laying eggs, the total brood size showed no significant difference between the group treated with ellagic acid and the control group (Figure 5B). Similar to the results for lifespan, 25 μM ellagic acid increased the motility of the nematodes, but 50 and 100 μM ellagic acid did not show a significant difference versus the control group worms on days 9, 13, and 17 (Figure 5C). Overall, all of these data suggest that ellagic acid cannot have a harmful effect on the health indexes of worms but can benefit worms at a relatively low concentration of 25 μM.

## 3. Discussion

Due to the destruction of environmental conditions, the ozone layer in the atmosphere is becoming thinner, and ultraviolet rays cannot be effectively absorbed. Ultraviolet rays partly reach the ground and result in various harmful effects on humans. Numerous studies have suggested that natural compounds derived from nature reduce the damage induced by ultraviolet radiation [30,31,32]. Ellagic acid is a natural polyphenol found in fruits such as pomegranates and red raspberries. Ellagic acid can alleviate the injury caused by UV radiation in human skin cell lines and mice [33,34]. In the present study, using nematodes as a model, the action of ellagic acid in improving stress damage was investigated. We found that ellagic acid significantly extended the mean lifespan of those nematodes exposed to UV radiation stress compared to the control group (Figure 1A). In addition, we also determined the effect of ellagic acid on the nematodes from various stressors. When subjected to 35 °C heat stress, the average lifespan of the worms was prolonged with ellagic acid treatment (Figure 1B). Under H_2_O_2_-induced oxidative stress and *Pseudomonas aeruginosa* infection, ellagic acid could also significantly prolong the average lifespan of the nematodes versus the control group (Figure 1C,D). Among these concentrations, 50 μM ellagic acid more obviously enhanced resistance in the nematodes afflicted by these stresses. These data suggest that ellagic acid can increase the stress resistance of nematodes under various stress conditions (UV radiation stress, heat stress, oxidative stress, and *Pseudomonas aeruginosa* infection stress).

We suggest that ellagic acid might activate the genes related to the stress response to increase the resistance to UV radiation in worms. Therefore, the expression of several genes involved in the stress response in nematodes exposed to UV radiation was determined. The expression of *daf-16* and *sod-3* was significantly upregulated in the worms treated with ellagic acid (Figure 2A). Moreover, ellagic acid could also upregulate the level of small heat shock protein genes (*hsp-16.1*, *hsp-16.2*, and *hsp-12.6*) in the worms exposed to UV radiation compared to the control group (Figure 2B).

DAF-16 is an important transcription factor related to the longevity and stress response in *C. elegans* [35,36,37]. Under normal conditions, DAF-16 is located in the cytoplasm but translocated to the nucleus to activate a series of genes and proteins involved in the stress response pathways to resist unfavorable factors under harmful conditions [23,38]. We found that ellagic acid significantly induced DAF-16 translocation from the cytoplasm to the nucleus in the transgenic worm strain TJ356 under UV radiation stress (Figure 3A,B). Moreover, the ellagic acid treatment also significantly upregulated the expression of SOD-3, a DAF-16 downstream protein in the CF1553 nematodes under UV radiation stress (Figure 3D,E). UV radiation can cause a large amount of ROS in organisms, which results in an oxidation reaction and accelerates the aging of organisms [39,40,41]. In this study, ellagic acid proved to significantly reduce the ROS content in the nematodes exposed to UV radiation (Figure 3G). As an endogenous antioxidant enzyme of organisms, SOD can remove harmful ROS produced by the body [42]. Via a SOD activity assay, ellagic acid treatment was shown to also increase the SOD activity (Figure 3H), which could catalyze the decomposition of superoxide free radicals and, thereby, efficiently fight the oxidative stress reaction in the worms exposed to UV radiation.

The insulin/IGF-1 signaling pathway is an important pathway to regulate lifespan, growth, development, metabolism, and stress in nematodes [43,44]. DAF-2 and DAF-16 are important components of the insulin/IGF-1 signaling pathway [26]. Therefore, we determined the effects of ellagic acid on *daf-16* mutant worms (CF1308) and *daf-2* and *daf-16* double-mutant worms (CF1588) exposed to UV radiation. Ellagic acid did not extend the average lifespan of the CF1308 and CF1588 nematodes under UV radiation (Figure 4A,B), which suggests that ellagic acid can resist UV radiation stress in nematodes through the insulin/IGF-1 signaling pathway.

Lifespan, fertility, and movement capacity are important health indexes in *C. elegans*. We also determined the effects of ellagic acid on the health indexes of nematodes, showing that 25 μM ellagic acid could significantly prolong the mean lifespan of the wild-type nematodes N_2_, while 50 and 100 μM ellagic acid did not have a significant effect on the lifespan of nematodes versus the control group (Figure 5A). However, the total brood size of the nematodes showed no differences between those worms treated with ellagic acid and the control worms (Figure 5B). Furthermore, 25 μM ellagic acid also increased the motility of the nematodes in the aging stages but 50 and 100 μM ellagic acid did not affect the movement capacity of the worms on days 9, 13, and 17 (Figure 5C). Overall, ellagic acid did not have adverse effects on the health indexes of the worms.

## 4. Materials and Methods

### 4.1. Preparation of Ellagic Acid

Ellagic acid (MedChemExpress, Monmouth Junction, NJ, USA) was dissolved in dimethyl sulfoxide (DMSO) and prepared as DMSO stock solutions of 0.5, 1.25, 2.5, 5, and 10 mM [45]. The stock solution was sterilized by filtration through a membrane with a 0.2 μm pore size. Subsequently, ellagic acid stock solutions were added to bacterial fluid (*Escherichia coli* OP50) to achieve final concentrations of 10, 25, 50, 100, and 200 μM. The mixture was then coated onto nematode growth medium (NGM) plates.

### 4.2. C. elegans Strains and Culture

Worm strains Bristol N_2_ (wild-type), TJ356 (zIs356[*daf-16p*::*daf-16a*/b::GFP + *rol-6*(*su1006*)]), CF1553 (muIs84[(pAD76) *sod-3p*::GFP + *rol-6*(*su1006*)]), CF1588 (*daf-16*(mu86) I; *daf-2*(e1370) III; muIs84), and CF1308 (*daf-16*(mu86) I) were obtained from the Caenorhabditis Genetic Center (University of Minnesota, Minneapolis, MN, USA).

The worms were cultured on nematode growth medium (NGM) agar plates containing *Escherichia coli* OP50 at 20 °C according to the standard protocol described previously [46].

### 4.3. Lifespan Assay

To synchronize the growth stage of *C. elegans*, N_2_ nematodes in gravid periods were transferred onto the NGM agar plates and their synchronized eggs were cultured at 20 °C until the L4-stage. The L4-stage worms were transferred onto NGM agar plates containing different concentrations of ellagic acid and, subsequently, transferred onto corresponding new plates every day. The survival of the worms was monitored every day until all of the worms died. *C. elegans* were judged to be dead if they could not move after being slightly touched with eyebrow hairs. A lifespan assay was performed three times (at least 100 worms per group).

### 4.4. Measurement of Egg-Laying Capacity

The synchronized L4-stage worms were transferred onto NGM agar plates containing different concentrations of ellagic acid. Each nematode was transferred onto a corresponding new plate every day until they no longer laid eggs. All of the eggs of each worm were added together. An assay of the laid eggs was conducted three times, and each group contained at least 10 worms.

### 4.5. Measurement of Movement Capacity

The synchronized L4-stage nematodes were transferred onto a culture on NGM agar plates containing different concentrations of ellagic acid. The motility of *C. elegans* was observed on days 9, 13, and 17, respectively. The movement state of *C. elegans* was divided into A, B, C, and D, and the movement state was judged as follows: A—*C. elegans* can move autonomously; B—*C. elegans* can move under slight touch; C—*C. elegans* can only move the head under mild touch; D—*C. elegans* cannot move, even when touched. An assay of movement capacity was conducted three times, and each group contained at least 100 *C. elegans*.

### 4.6. Stress Resistance Assay

Pregnant N_2_ worms were transferred onto NGM agar plates to synchronize their growth stage. Synchronized eggs were developed at 20 °C until the L4-stage. The L4-stage worms were transferred onto NGM agar plates containing different concentrations of ellagic acid for two days and used for the following stress resistance assay. For the heat stress assay, the culture temperature of the worms was adjusted from 20 °C to 35 °C and the survival was recorded every 1 h until all worms died. For the UV radiation assay, the worms were continuously exposed to UV radiation of 254 nm at a dose of 1000 J/cm^2^ and transferred onto new plates every day. Then, the survival of the nematodes was observed every 12 h until all the nematodes died. As for the oxidative stress assay, the worms were transferred onto 96-well plates containing 200 μL of S medium with a final concentration of 0.4 mM H_2_O_2_ (Chemical Industry Group, Beijing, China) [47]. Survival of the worms was observed every 0.5 h until all worms died. Finally, a *Pseudomonas aeruginosa* infection assay was conducted by transferring nematodes onto NGM agar plates containing *Pseudomonas aeruginosa*, and their survival was recorded every 1 h until all nematodes died.

### 4.7. Measurement of Reactive Oxygen Species (ROS)

The L4-stage worms were transferred onto NGM agar plates with or without ellagic acid for two days and subsequently transferred onto new NGM plates and exposed to UV radiation of 254 nm at a dose of 1000 J/cm^2^ at 20 °C. After 12 h, the nematodes were collected and loaded with 10 µM DCFH-DA (Beyotime, Shanghai, China) dissolved in M9 buffer (42 mM Na_2_HPO_4_, 22 mM KH_2_PO_4_, 85.5 mM NaCl, and 1 mM MgSO_4_) for 1 h in the dark. Afterward, the nematodes were washed with M9 buffer three times and transferred onto dark 96-well plates containing 200 μL of M9 buffer. The fluorescence intensity at an excitation wavelength of 485 nm and an emission wavelength of 535 nm was measured using a microplate reader (Infinite 200 PRO, TECAN, Zürich, Switzerland).

### 4.8. Assay of Superoxide Dismutase (SOD) Activity

The L4-stage worms were transferred onto NGM agar plates with or without ellagic acid for two days. Then, the worms were transferred onto new NGM plates and exposed to UV radiation for 12 h. Afterward, the worms were collected with M9 buffer containing protease inhibitor and sonicated on ice. The SOD activity of the extracted protein was measured via a microplate reader at a wavelength of 560 nm using a SOD activity assay kit (Solarbio, Beijing, China).

### 4.9. Nucleus Localization Analysis of DAF-16

The young adult TJ356 nematodes were cultured on NGM agar plates with or without ellagic acid for two days. After this, the TJ356 nematodes were exposed to UV radiation of 254 nm at a dose of 1000 J/cm^2^ and, subsequently, transferred onto new NGM agar plates to culture for 12 h at 20 °C. The nematodes were anesthetized with 50 mM sodium azide and placed on a glass slide covered with 5% agar. The images were captured using a fluorescence microscope (IX73, Olympus, Tokyo, Japan) and the amount of DAF-16 in the nucleus was measured with ImageJ software (V1.8.0.172, National Institutes of Health, Bethesda, MD, USA).

### 4.10. Quantitative Real-Time PCR (qPCR)

The L4-stage worms (N_2_) were transferred onto NGM agar plates with or without ellagic acid for two days. Then, the worms were exposed to UV radiation of 254 nm at a dose of 1000 J/cm^2^ and transferred onto new NGM agar plates. After being cultured at 20 °C for 12 h, the worms were collected with M9 buffer and lysed to extract RNA with Trizol (TransGen, Beijing, China), and their cDNA was synthesized using TransScript One-Step gDNA Removal and cDNA Synthesis SuperMix (TransGen, Beijing, China). Afterward, qPCR was performed with TransStart Top Green qPCR SuperMix (TransGen, Beijing, China) using a PCR instrument (ABI7500, Applied Biosystems, Waltham, MA, USA) with *act-1* (actin) as the reference gene. The ΔΔCT method was used to analyze the difference in gene expression. The specific gene primers are listed in Table 1.

### 4.11. Measurement of SOD-3 Expression in the Nematodes

The young adult CF1553 worms were transferred onto NGM agar plates with or without ellagic acid for two days and exposed to UV radiation of 254 nm at a dose of 1000 J/cm^2^. After being transferred onto new NGM agar plates for 12 h, the CF1553 worms were anesthetized with 50 mM sodium azide and put onto a glass slide covered with 5% agar. Photos were taken using a fluorescence microscope and the fluorescence intensity was determined using ImageJ software (at least 15 nematodes per group).

### 4.12. UV Radiation Assay of Mutant C. elegans Strains

The young adult CF1588 and CF1308 worms were transferred onto NGM agar plates with or without ellagic acid for two days. After being exposed to UV radiation of 254 nm at a dose of 1000 J/cm^2^, the CF1588 and CF1308 worms were transferred onto new NGM agar plates. The survival of the nematodes was observed every 12 h until all worms died. Three independent experiments were conducted (at least 100 worms per group).

### 4.13. Statistical Analysis

Statistical analysis was performed with GraphPad Prism (GraphPad Software, V8.0.1, La Jolla, CA, USA). Log-rank (Mantel-Cox) survival analyses were used for the lifespan and stress-resistance experiments. Comparisons between the control and treatment *C. elegans* were conducted using one- or two-way ANOVA. The *p*-value represents any statistical differences, with *p* < 0.05 (*), *p* < 0.01 (**), and *p* < 0.001 (***) being regarded as significantly different.

## 5. Conclusions

In conclusion, ellagic acid increased the resistance of nematodes under various stressors (UV radiation stress, heat stress, oxidative stress, and *Pseudomonas aeruginosa* infection stress). Ellagic acid increased the UV radiation stress resistance in nematodes, probably via activating the genes related to the stress response; i.e., *daf-16*, *sod-3, hsp-16.1*, *hsp-16.2*, and *hsp-12.6.* Moreover, ellagic acid could significantly induce DAF-16 translocation from the cytoplasm to the nucleus, reduce the ROS content, and increase the SOD activity in worms exposed to UV radiation. Finally, ellagic acid increased the resistance to UV radiation stress of nematodes, probably in an insulin/insulin-like growth factor-1 (IGF-1) signaling pathway-dependent manner. This provides a theoretical basis for ellagic acid as a possible drug candidate for the treatment of stress-related diseases and also promotes research on and the industrial development of natural products with market prospects.

## Figures and Tables

**Table 1:** Specific genes primers.

Genes	Forward Primers	Reverse Primers
*act-1*	5′-CCAGGAATTGCTGATCGTATGCAGAA-3′	5′-TGGAGAGGGAAGCGAGGATAGA-3′
*daf-16*	5′-TTTCCGTCCCCGAACTCAA-3′	5′-ATTCGCCAACCCATGATGG-3′
*sod-3*	5′-TTCGAAAGGGAATCTAAAAGAAG-3′	5′-GCCAAGTTGGTCCAGAAGATAG-3′
*hsf-1*	5′-TTGACGACGACAAGCTTCCAGT-3′	5′-AAAGCTTGCACCAGAATCATCCC-3′
*hsp-16.2*	5′-CTGCAGAATCTCTCCATCTGAGTC-3′	5′-AGATTCGAAGCAACTGCACC-3′
*hsp-16.49*	5′-GTCAAATCTGCAATTTCGAATG-3′	5′-CAAAATTAATGGGAATAGAACGAG-3′
*hsp-12.6*	5′-TGGCCACTTCAAAAGGGAG-3′	5′-CTCTTTTGGGAGGAAGTTATGG-3′

## Data Availability

Not applicable.

## References

[1] Li S., Liu D., Liu Y., Liu B., Chen X. (2020). Quercetin and its mixture increase the stress resistance of *Caenorhabditis elegans* to UV-B. Int. J. Environ. Res. Public Health.

[2] Ball D. (2021). Contrasting effects of heat stress on neuromuscular performance. Exp. Physiol..

[3] Tan B.L., Norhaizan M.E., Liew W.P. (2018). Nutrients and oxidative stress: Friend or foe?. Oxid. Med. Cell Longev..

[4] Slimen I.B., Najar T., Ghram A., Dabbebi H., Ben Mrad M., Abdrabbah M. (2014). Reactive oxygen species, heat stress and oxidative-induced mitochondrial damage. Int. J. Hyperth..

[5] Matsumura Y., Ananthaswamy H.N. (2004). Toxic effects of ultraviolet radiation on the skin. Toxicol. Appl. Pharmacol..

[6] Narayanan D.L., Saladi R.N., Fox J.L. (2010). Ultraviolet radiation and skin cancer. Int. J. Dermatol..

[7] Mullenders L.H.F. (2018). Solar UV damage to cellular DNA: From mechanisms to biological effects. Photochem. Photobiol. Sci..

[8] Benz C.C., Yau C. (2008). Ageing, oxidative stress and cancer: Paradigms in parallax. Nat. Rev. Cancer.

[9] Saewan N., Jimtaisong A. (2015). Natural products as photoprotection. J. Cosmet. Dermatol..

[10] Cavinato M., Waltenberger B., Baraldo G., Grade C.V.C., Stuppner H., Jansen-Dürr P. (2017). Plant extracts and natural compounds used against UVB-induced photoaging. Biogerontology.

[11] Sharifi-Rad J., Quispe C., Castillo C.M.S., Caroca R., Lazo-Vélez M.A., Antonyak H., Polishchuk A., Lysiuk R., Oliinyk P., De Masi L. (2022). Ellagic acid: A review on its natural sources, chemical stability, and therapeutic potential. Oxid. Med. Cell Longev..

[12] Zeb A. (2018). Ellagic acid in suppressing in vivo and in vitro oxidative stresses. Mol. Cell Biochem..

[13] Derosa G., Maffioli P., Sahebkar A. (2016). Ellagic acid and its role in chronic diseases. Adv. Exp. Med. Biol..

[14] De R., Sarkar A., Ghosh P., Ganguly M., Karmakar B.C., Saha D.R., Halder A., Chowdhury A., Mukhopadhyay A.K. (2018). Antimicrobial activity of ellagic acid against Helicobacter pylori isolates from India and during infections in mice. J. Antimicrob. Chemother..

[15] Li Z.J., Guo X., Dawuti G., Aibai S. (2015). Antifungal activity of ellagic acid in vitro and in vivo. Phytother. Res..

[16] Azam F., Azam F., Khan M.A., Khan A., Ahmad S., Zofair S.F.F., Younus H. (2022). In silico and in vitro studies on the inhibition of laccase activity by ellagic acid: Implications in drug designing for the treatment of cryptococcal infections. Int. J. Biol. Macromol..

[17] Khan M.A., Khan A., Azam M., Allemailem K.S., Alrumaihi F., Almatroudi A., Alhumaydhi F.A., Azam F., Khan S.H., Zofair S.F.F. (2021). Liposomal ellagic acid alleviates cyclophosphamide-induced toxicity and eliminates the systemic cryptococcus neoformans infection in leukopenic Mice. Pharmaceutics.

[18] Moon N.R., Kang S., Park S. (2018). Consumption of ellagic acid and dihydromyricetin synergistically protects against UV-B induced photoaging, possibly by activating both TGF-β1 and wnt signaling pathways. J. Photochem. Photobiol. B.

[19] Baek B., Lee S.H., Kim K., Lim H.W., Lim C.J. (2016). Ellagic acid plays a protective role against UV-B-induced oxidative stress by up-regulating antioxidant components in human dermal fibroblasts. Korean J. Physiol. Pharmacol..

[20] Bae J.Y., Choi J.S., Kang S.W., Lee Y.J., Park J., Kang Y.H. (2010). Dietary compound ellagic acid alleviates skin wrinkle and inflammation induced by UV-B irradiation. Exp. Dermatol..

[21] Wang H., Liu J., Li T., Liu R.H. (2018). Blueberry extract promotes longevity and stress tolerance via DAF-16 in *Caenorhabditis elegans*. Food Funct..

[22] Muñoz M.J. (2003). Longevity and heat stress regulation in *Caenorhabditis elegans*. Mech. Ageing Dev..

[23] Henderson S.T., Johnson T.E. (2001). Daf-16 integrates developmental and environmental inputs to mediate aging in the nematode *Caenorhabditis elegans*. Curr. Biol..

[24] Sun X., Chen W.D., Wang Y.D. (2017). DAF-16/FOXO transcription factor in aging and longevity. Front. Pharmacol..

[25] Yanase S., Yasuda K., Ishii N. (2020). Interaction between the ins/IGF-1 and P38 MAPK signaling pathways in molecular compensation of sod genes and modulation related to intracellular ROS levels in *C. elegans*. Biochem. Biophys. Rep..

[26] Murphy C.T., Hu P.J. (2013). Insulin/insulin-like growth factor signaling in *C. elegans*. WormBook.

[27] Zhang S., Li F., Zhou T., Wang G., Li Z. (2020). *Caenorhabditis elegans* as a useful model for studying aging mutations. Front. Endocrinol..

[28] Mukhopadhyay A., Oh S.W., Tissenbaum H.A. (2006). Worming pathways to and from DAF-16/FOXO. Exp. Gerontol..

[29] Zečić A., Braeckman B.P. (2020). DAF-16/FoxO in *Caenorhabditis elegans* and its role in metabolic remodeling. Cells.

[30] Rajnochová Svobodová A., Ryšavá A., Čížková K., Roubalová L., Ulrichová J., Vrba J., Zálešák B., Vostálová J. (2022). Effect of the flavonoids quercetin and taxifolin on UVA-induced damage to human primary skin keratinocytes and fibroblasts. Photochem. Photobiol. Sci..

[31] Psotova J., Svobodova A., Kolarova H., Walterova D. (2006). Photoprotective properties of prunella vulgaris and rosmarinic acid on human keratinocytes. J. Photochem. Photobiol. B.

[32] De Silva W.G.M., Abboud M., Yang C., Dixon K.M., Rybchyn M.S., Mason R.S. (2020). Protection from ultraviolet damage and photocarcinogenesis by vitamin D compounds. Adv. Exp. Med. Biol..

[33] Hseu Y.C., Chou C.W., Senthil Kumar K.J., Fu K.T., Wang H.M., Hsu L.S., Kuo Y.H., Wu C.R., Chen S.C., Yang H.L. (2012). Ellagic acid protects human keratinocyte (HaCaT) cells against UVA-induced oxidative stress and apoptosis through the upregulation of the HO-1 and Nrf-2 antioxidant genes. Food Chem. Toxicol..

[34] Gorgisen G., Ozkol H., Tuluce Y., Arslan A., Ecer Y., Keskin S., Kaya Z., Ragbetli M.C. (2020). Silibinin and ellagic acid increase the expression of insulin receptor substrate 1 protein in ultraviolet irradiated rat skin. Biotech. Histochem..

[35] Cypser J.R., Tedesco P., Johnson T.E. (2006). Hormesis and aging in *Caenorhabditis elegans*. Exp. Gerontol..

[36] Wang W., Feng X., Du Y., Liu C., Pang X., Jiang K., Wang X., Liu Y. (2021). Synthesis of novel pinocembrin amino acid derivatives and their antiaging effect on *Caenorhabditis elegans* via the modulating DAF-16/FOXO. Drug Des. Devel. Ther..

[37] Tullet J.M.A. (2015). DAF-16 target identification in *C. elegans*: Past, present and future. Biogerontology.

[38] Hesp K., Smant G., Kammenga J.E. (2015). *Caenorhabditis elegans* DAF-16/FOXO transcription factor and its mammalian homologs associate with age-related disease. Exp. Gerontol..

[39] de Jager T.L., Cockrell A.E., Du Plessis S.S. (2017). Ultraviolet light induced generation of reactive oxygen species. Adv. Exp. Med. Biol..

[40] Wang M., Charareh P., Lei X., Zhong J.L. (2019). Autophagy: Multiple mechanisms to protect skin from ultraviolet radiation-driven photoaging. Oxid. Med. Cell Longev..

[41] Rinnerthaler M., Bischof J., Streubel M., Trost A., Richter K. (2015). Oxidative stress in aging human skin. Biomolecules.

[42] Wang Y., Branicky R., Noë A., Hekimi S. (2018). Superoxide dismutases: Dual roles in controlling ROS damage and regulating ROS signaling. J. Cell Biol..

[43] Martins R., Lithgow G.J., Link W. (2016). Long live FOXO: Unraveling the role of FOXO proteins in aging and longevity. Aging Cell.

[44] Altintas O., Park S., Lee S.J.V. (2016). The role of insulin/IGF-1 signaling in the longevity of model invertebrates, *C. elegans* and *D. melanogaster*. BMB Rep..

[45] Soh P.N., Witkowski B., Olagnier D., Nicolau M.L., Garcia-Alvarez M.C., Berry A., Benoit-Vical F. (2009). In vitro and in vivo properties of ellagic acid in malaria treatment. Antimicrob. Agents Chemother..

[46] Stiernagle T. (2006). Maintenance of *C. elegans*. WormBook.

[47] Solis G.M., Petrascheck M. (2011). Measuring *Caenorhabditis elegans* life span in 96 well microtiter plates. J. Vis. Exp..

